# Optical focal plane based on MEMS light lead-in for geometric camera calibration

**DOI:** 10.1038/micronano.2017.58

**Published:** 2017-11-06

**Authors:** Jin Li, Zilong Liu

**Affiliations:** 1 Department of Precision Instrument, Tsinghua University, Beijing 100084, China; 2 Optical Division, National Institute of Metrology, Beijing 100029, China

**Keywords:** camera calibration, MEMS light lead-in, robustness

## Abstract

The focal plane of a collimator used for the geometric calibration of an optical camera is a key element in the calibration process. The traditional focal plane of the collimator has only a single aperture light lead-in, resulting in a relatively unreliable calibration accuracy. Here we demonstrate a multi-aperture micro-electro-mechanical system (MEMS) light lead-in device that is located at the optical focal plane of the collimator used to calibrate the geometric distortion in cameras. Without additional volume or power consumption, the random errors of this calibration system are decreased by the multi-image matrix. With this new construction and a method for implementing the system, the reliability of high-accuracy calibration of optical cameras is guaranteed.

## Introduction

High-accuracy determination of the position of features in the field of view (positioning) is one of the most significant functions of a camera used on a satellite. Knowledge of the inner orientation parameters (IOPs) of an optical camera, such as its principal distance and principal point, is a prerequisite of image positioning^1–4^. The accuracy of camera calibration strongly influences the accuracy in determining image position. After developing a camera, calibrations of the camera IOPs are necessary.

Many approaches to calibrating the IOPs of optical cameras exist^5–14^. For a remote sensing camera, the main calibration method is based on measuring angles to known points to determine the principal distance and the principal point of the optics^15,16^. In this method, an uncalibrated optical camera is placed on a precision turntable to capture the parallel rays of light of star points emitted by a collimator. The camera can obtain multiple positions of the star points and their images in different fields of view (FOVs) as the turntable is rotated. The recorded rotation angle and image coordinates are used to construct an equation that relates each star point to its corresponding location in the image. On the basis of an analysis of camera distortion, the optical camera has its minimum distortion at the location of its principal plane^17–19^. A least squares method is used to solve the imaging equations to calculate the principal distance and the location of the principal point. In this method, a single-hole light lead-in (SHLL) is positioned at the focal plane of the collimator. The measured results using the SHLL only represent one area of the FOV. To acquire imaging information for the entire FOV, a turntable is needed to access all the necessary points in the FOV. However, multiple sources of error at every rotation angle, such as turntable vibration, angle measurement errors and image positioning errors, may influence the measurement result. In accessing the full FOV, the accumulated error may influence the calibration results. As a result, the calibration accuracy is relatively unreliable.

In this study, we propose and evaluate a micro-electro-mechanical system light lead-in array device (MEMS-LLAD) composed of a porous silicon array; this system is located at the optical focal plane of the collimator used to calibrate an optical remote sensing camera. The result of using the MEMS-LLAD represents the area of one FOV. The rotation time is decreased when acquiring the full FOV, enabling a reduction in measurement error. Moreover, the accuracy in extracting point positions can also be improved because of the multiple aperture structures of the MEMS-LLAD.

## Materials and methods

### The calibration principle of using a light lead-in collimator

**Figure 1:**
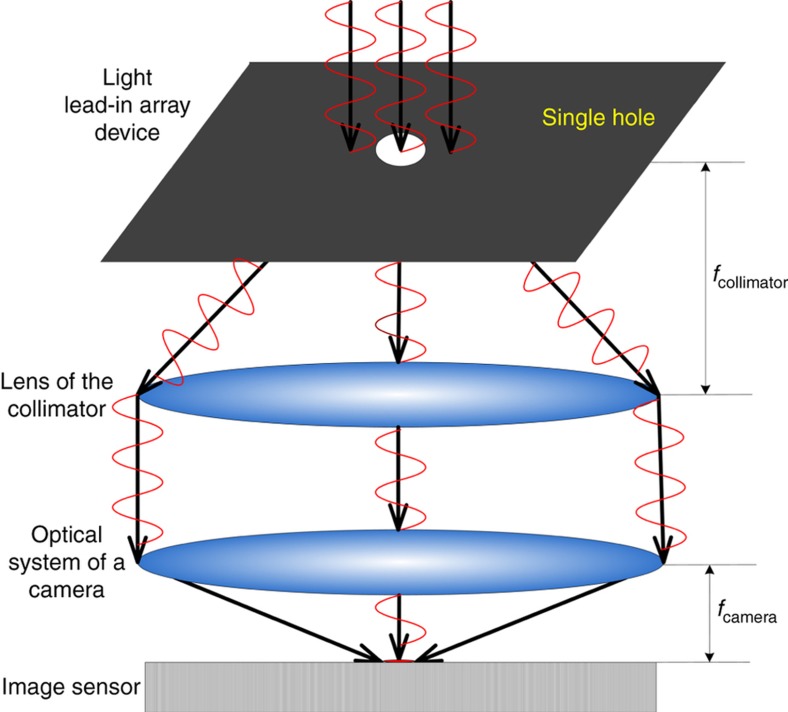
Sketch of the single-hole optical focal plane.

A traditional collimator typically uses a single structure, as shown in Figure 1. Calibration is performed by means of a turntable. From different turntable rotation angles, the camera obtains multiple positions for the star point in different FOVs.

**Figure 2:**
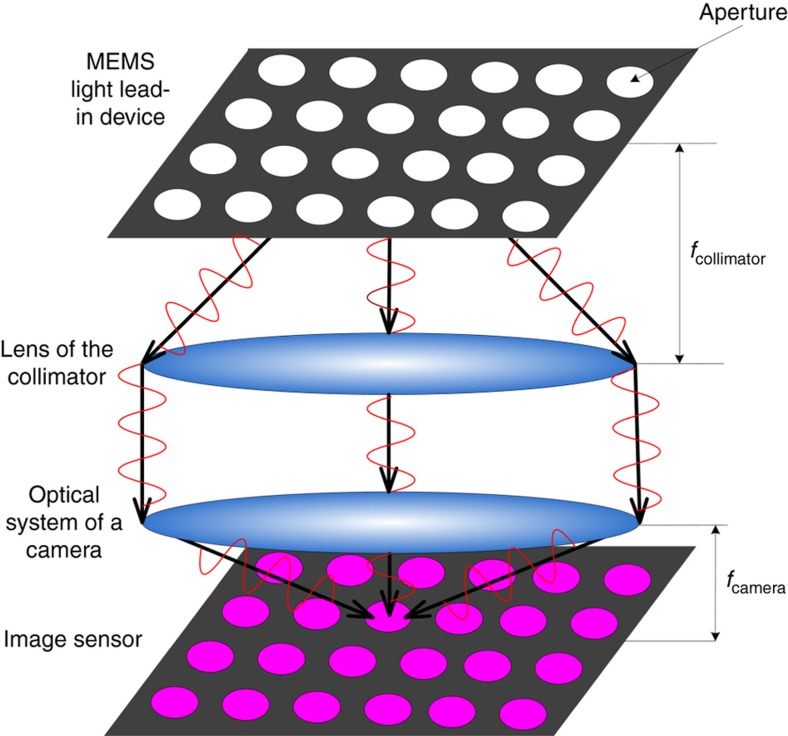
Sketch of multiple hole optical focal plane array.

On the basis of the distortion theory of an optical camera and the geometric imaging relationship, a least squares method can be used to calculate the principal distance and the principal point, which can be expressed as^17–20^:

(1)f=n×∑i=1n(yi×tanωyi)−∑i=1ntanωyi∑i=1nyin×∑i=1ntan2ωyi−(∑i=1ntanωyi)2

(2)(x0,y0)=([∑i=1nyi−(∑i=1ntanωyi)×f]n,[∑i=1nxi−(∑i=1ntanωxi)×f]n)

where (*x*_*i*_,*y*_*i*_) is the coordinate position of the image of the hole in different FOVs in the image space. (*x*_0_,*y*_0_) is the coordinate of the principal point. $\left(\omega_{x}^{i},\omega_{y}^{i}\right)$ is the angle of the FOV in the *x* and *y* directions. On the basis of Equations (1) and (2), (*x*_*i*_, *y*_*i*_) is the measured position of the image point, and $\left(\omega_{x}^{i},\omega_{y}^{i}\right)$ is determined by the turntable. The error of the turntable can be as large as $\delta_{\omega }=0.5\rho /\sqrt{3}=0.29\rho =1.405\times 10^{-6}$, where ρ is 1/3600 degrees. $\delta_{\omega }=0.5\prime \prime /\sqrt{3}=0.29\prime \prime =1.405\times 10^{-6}$. The calibration accuracy depends mainly on the positional accuracy of the measured image coordinates (*x*_*i*_,*y*_*i*_). However, the position measurement accuracy when using an SHLL is easily influenced by multiple sources of noise. These errors will result in low calibration accuracy. To address this problem, we propose using multiple aperture arrays to calibrate remote sensing cameras. The structure of the multiple aperture array is shown in Figure 2.

When an MEMS-LLAD is used to perform camera calibration, the image acquired at every rotation angle has multiple image points. Each image point results in a measured point in the FOV. The MEMS-LLAD can cover multiple points in the FOV at every rotation angle. In the MEMS-LLAD, each aperture is encoded and accurately positioned. On the basis of the camera model and the mapping of three-dimensional object coordinates to two-dimensional image coordinates, when ideal rays from the MEMS-LLAD are projected onto the image plane, the ideal pixel image coordinates of the *i*-th image point are expressed by:

(3)[uivi1]=[f0u00fv0001][x′iy′i1]

where

(4)[x′iy′i1]=[xifc+tanωxtyifc+tanωyt1]

and [*x*_*i*_,*y*_*i*_]^T^ are the encoded coordinates of the *i*-th aperture in the MEMS-LLAD, ${\left[{x\prime }_{i},{y\prime }_{i}\right]}^{T}$ are the ideal normalized image coordinates, *f*_*c*_ is the focal length of the collimator, *f* is the principal distance, [*u*_0_,*v*_0_]^T^ is the principal point and $\left(\omega_{x}^{t},\omega_{y}^{t}\right)$ is the rotation angle of the turntable in *x* and *y* directions at *t* time. When lens distortion is considered, the model can be expanded by inserting a term to account for the distortion position error *ε*,

(5)[x′ˆiy′ˆi]=[x′iy′i]+ε(x′i,y′)

where

(6)ε(x′i,y′)=[x′iy′i](k1r2+k2r4+k3r6)

and $r^{2}={\left({x\prime }_{i}\right)}^{2}+{\left(y\prime \right)}^{2}$. The final mapping of ideal points to distorted image coordinates ${\left[\hat{u},\hat{v}\right]}^{T}$ can be expressed as:

(7)[uˆivˆi]=[u0v0]+f[x′iy′i](k1r2+k2r4+k3r6)

Using a set of image points and rotation angles, we can solve the following cost function to calculate the IOPs:

(8)min‖[uˆi−u0vˆi−v0]−f[xifc+tanωxtyifc+tanωyt](k1r2+k2r4+k3r6)‖2

**Figure 3:**
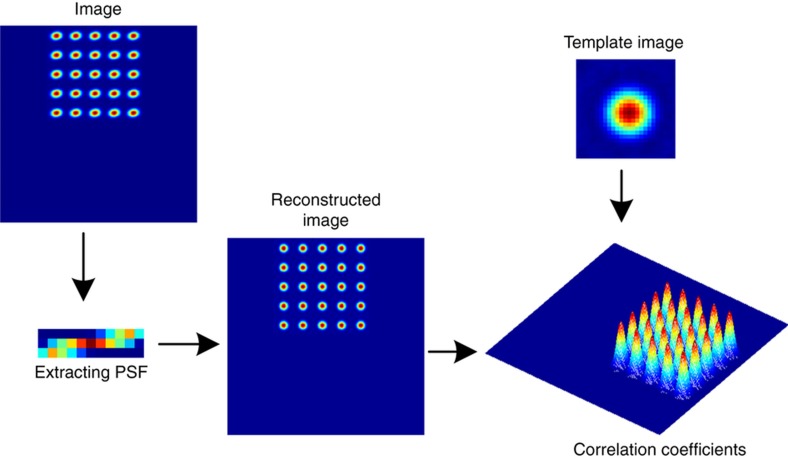
The position extraction algorithm. PSF: point spread function.

To accurately determine the coordinates ${\left[\begin{array}{cc} {\hat{u}}_{i} & {\hat{v}}_{i} \end{array}\right]}^{T}$ in Equation (8), we utilized multiple image points, correcting the optical aberrations caused by vibration, system errors and extraction errors, to calibrate the camera. Figure 3 shows the principle of position extraction. First, we extracted an array template from the MEMS-LLAD image at its original angle. Second, multiple image points at every rotated angle were used as sub-resolution features (SRFs) to invert the point spread function (PSF) using a sparse total variation frame^21^. The reconstructed PSF was used to reconstruct a high-resolution image to correct for optical aberrations^22^. We used the extracted template to perform cross-correlation with the images captured at the different rotation angles. The correlation operation can be expressed as:

(9)C(m,n)=∑i=0L∑j=0LP1(m+i,n+j)P0(i,j)

where *P*_0_(*i*,*j*) is the extracted template image. We used a centroid method to extract the position of each image point from the correlation value.

The centroid value is calculated by the following equation:

(10)x=∑i=110∑j=110m×C(m,n)∑i=110∑j=110C(m,n),y=∑i=110∑j=110n×C(m,n)∑i=110∑j=110C(m,n)

Equation (4) can be used to calculate the accurate position at a certain FOV angle (FOVA). On the basis of Equations (1) and (2), the principal distance and principal point can be calculated.

### Fabrication of MEMS light lead-in array device

**Figure 4:**
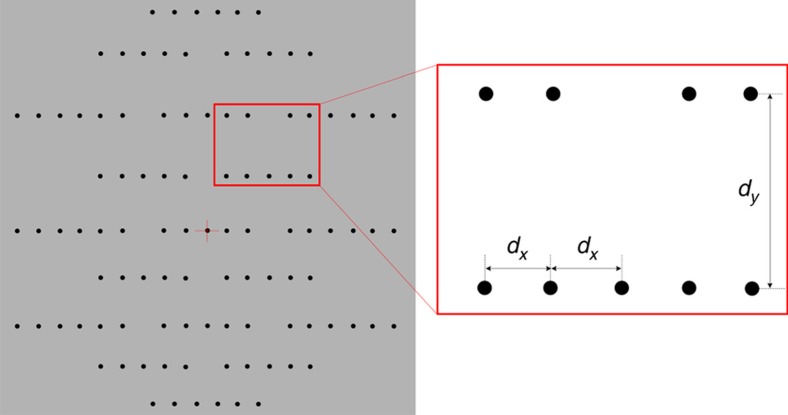
Encode aperture in light lead-in array device.

The size of a multi-hole MEMS-LLAD is mainly determined by the size and resolution of the image sensor of the camera. In our uncalibrated camera, the active area of the image sensor is 5.42 mm×6.78 mm. The pixel size is 5.3 μm×5.3 μm. The focal length of the camera and collimator is 2 m and 1.8 m, respectively. The active area of the LLAD is 4.878 mm×6.102 mm. The minimum size of each hole is 4.77 μm×4.77 μm. The LLAD pattern we designed is shown in Figure 4.

**Figure 5:**
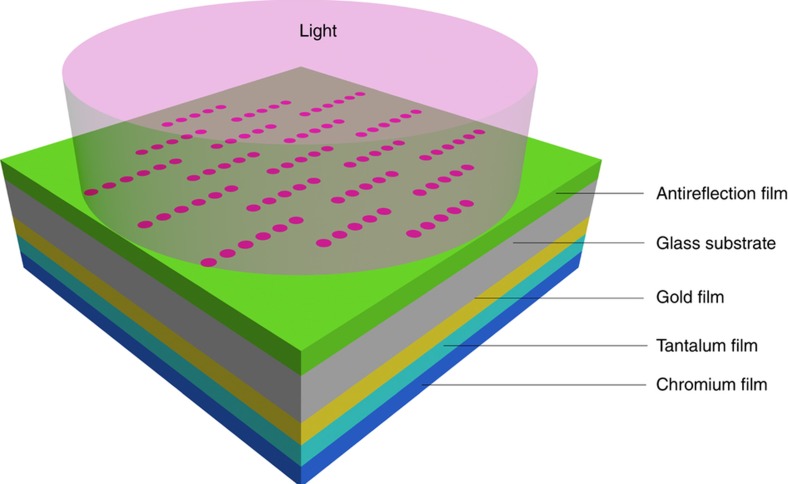
Schematic of the light lead-in array device structure.

Figure 5 shows a schematic of the LLAD structure. The entire structure is a stack. Between the surface on which light is incident to the exit surface, there is an antireflection film, neutral-tinted glass substrate, mask layers and a secondary anti-reflection layer. The neutral-tinted glass is used as the coating substrate. Optical anti radiation quartz glass is used as the glass substrate. The mask layers are composed of gold film and tantalum film. The chromium film is used as a secondary antireflection layer.

**Figure 6:**
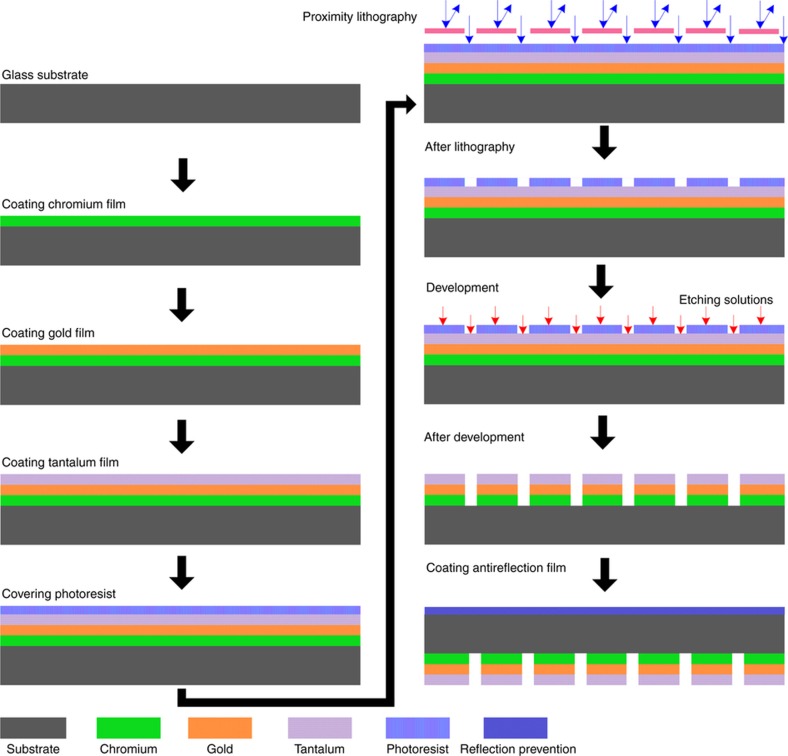
MEMS process for fabricating a point-source focal plane. MEMS: micro-electro-mechanical system.

Using MEMS machining technology, we established a machining process to fabricate the LLAD used in this work. The fabrication process is shown in Figure 6 and can be summarized in two steps: (1) chromium, gold and tantalum materials are plated on a specified glass substrate; and (2) a photoetching process is performed on the metal layers to create several small apertures. The LLAD includes a glass substrate, a mask layer and an anti-reflective layer; the mask thickness is 1.6 mm. The etched apertures in the mask let light pass through, whereas other parts cannot because they are covered by metal layers. First, a layer of chromium is plated on the glass substrate. The layer of chromium completely attenuates the light with its thickness of 75 nm, which effectively determines the transmissivity of the optical system. Second, the gold membrane is plated on the chromium layer. The gold membrane is a mask layer with a thickness of 200 nm. Third, the tantalum membrane is plated on the gold layer and is a radiation protection layer; its thickness is 60 nm. Fourth, the photoresist is deposited onto the plated substrate by spin coating. A polymethylmethacrylate material is used as the photoresist in the fabrication process. Fifth, proximity lithography is performed to expose the photoresist through the photomask. Sixth, developer is applied to remove the exposed photoresist to form the pattern on the plated substrate. Seventh, a second chromium layer is plated on the cut substrate. This layer is used as the secondary antireflection layer; its thickness is 75 nm. The number of holes in the MEMS LLAD influences system accuracy. On the basis of random errors, the precision of a multi-aperture system is $1/\sqrt{N}$ of the single aperture system, when the number of apertures is *N*.

## Results

**Figure 7:**
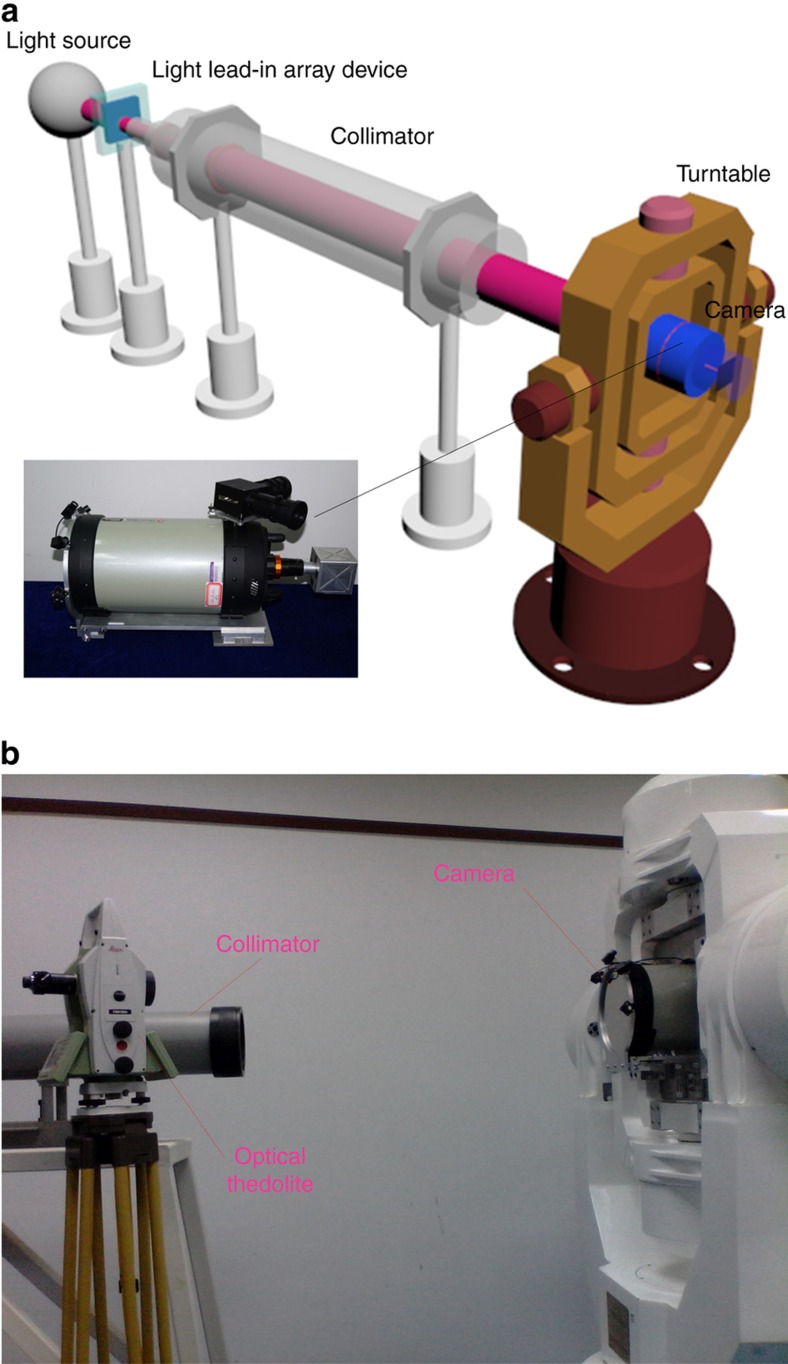
Experimental system: (**a**) the schematic diagram, and (**b**) the actual set-up.

A customized camera was developed to verify the proposed method, and the MEMS-LLAD was used to set up the experimental system. Figure 7 shows the experimental arrangement. The system included an optical camera, an MEMS-LLAD, a collimator and a high-accuracy turntable. The MEMS-LLAD was installed at the focal plane of the collimator. The collimator provided a target at infinity for the test system. The remote sensing camera is composed of an optical system, an image sensor and a processing circuit. The optical system was a co-axial Schmidt–Cassegrain optical system. By design, the aperture diameter was 203.2 mm, and the focal length was 2032 mm; hence the F/ratio of the optical system was 10. The image sensor was a CMOS detector with an image resolution of 1280×1024 pixels. In the process of adjusting the system, several pieces of auxiliary equipment, such as an optical theodolite and benchmark prisms, were utilized to determine the relative positions of the remote sensing camera and the MEMS-LLAD.

**Figure 8:**
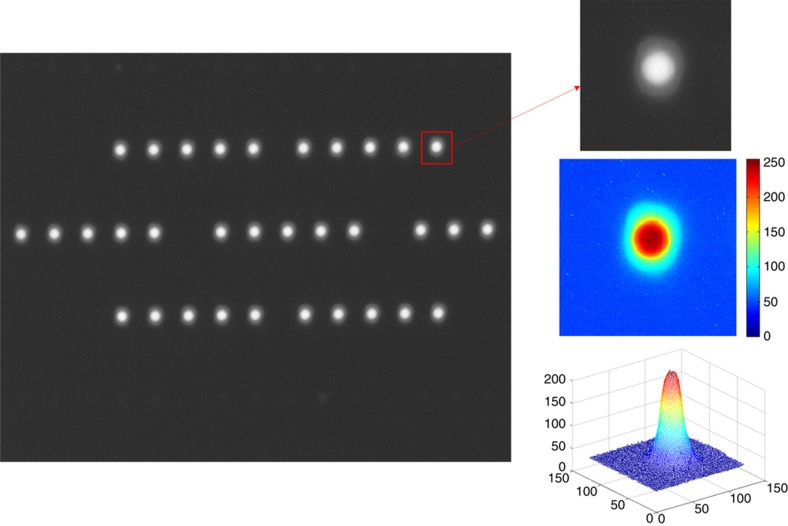
Captured images of micro-electro-mechanical system light lead-in array device.

The camera controller set the imaging mode. The three-axis turntable was adjusted evenly using the optical theodolite. The collimator was also adjusted evenly. By adjusting the support tooling of the camera and using the benchmark prisms located on the camera’s optical axis, the camera’s visual axis and collimator were adjusted to share a common axis. The MEMS-LLAD in the collimator was imaged onto the target CMOS sensor. Figure 8 shows the captured images.

**Figure 9:**
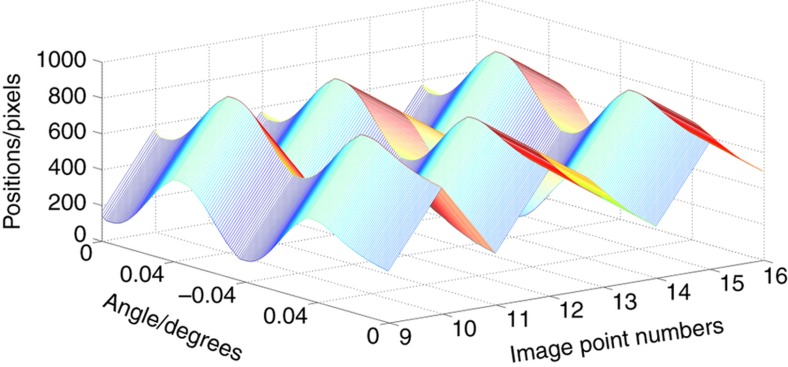
Position trajectory.

As the turntable was rotated, the rotation angle was recorded along with the captured images. The processing circuitry output the star coordinates in real time. Figure 9 shows the position trajectories of the image points in one period.

Least squares two-multiplication regression analysis was used to obtain the optimal estimated values of the IOPs based on the measured centroid positions and the rotation angles. In addition, the SHLL was used to calibrate the IOPs of the camera for reference. Table 1 shows the results obtained using the two methods.

In our experimental process, the MEMS-LLAD was used to calibrate the camera under the same conditions as that for using the SHLL method. We used vibration isolation and maintained a constant room temperature to suppress any environmental influence and provide a consistent imaging environment for the two methods. On the basis of Equation (1), the errors can be expressed as:

(11)δf=(∑i=1n(∂f∂yi)2δy2+∑i=1n(∂f∂ωi)2δω2)12=(Fyδy2+Fωδω2)12

(12)δy0=(∑i=1n(∂y0∂yi)2δy2+∑i=1n(∂y0∂ωi)2δω2+∑i=1n(∂y0∂f)2δf2)12=(Pyδy2+Pωδω2+Pfδf2)12

where *δ*_*y*_ are the position measurement errors, which depend on the accuracy of the extraction algorithm, *δ*_*y*_ are the angle measurement errors, *F*_*y*_ are the transfer parameters of the centroid displacement errors for the image points and *F*_*ω*_ are the transfer parameters of the angle measurement errors. In the SHLL measurement, the centroid extraction accuracy can reach 1/10 of a pixel and *δ*_*y*_=5.3 μm/10=0.53 μm. When using the MEMS-LLAD, we used cross-correlation to extract the point positions. Generally, the extraction accuracy when using cross-correlation can reach 0.01–0.07 pixel^23,24^. In our method, we utilized the SRF inversion method to correct the optical aberrations. The position measurement errors using this method are smaller than 0.01 pixel, which means ${\delta_{y}}^{MEMS-LLAD}<{\delta_{y}}^{SHLL}$. Because the number of turntable positions when using this method is smaller than when using the SHLL, *F*_*ω*_ and *F*_*y*_ are smaller than when using the SHLL. Therefore, the errors of the IOPs when using this method are reduced than when using the SHLL, which is consistent with the experimental results seen in Table 1. On the basis of the above-mentioned analysis, the MEMS-LLAD method has the merits in the position extraction accuracy and the error transfer parameters, enabling improved calibration precision.

## Discussion

In the experimental set-up, the MEMS-LLAD may be affected by turntable vibrations, environmental vibrations, temperature and airflows, leading to errors in calibration. The pattern of the MEMS-LLAD itself may influence the calibration accuracy. The greater the number of apertures used, the less the rotation number and the higher the calibration accuracy that can be obtained. However, a larger number of apertures will result in difficulties with fabrication. On the basis of the SRF inversion algorithm, a stable PSF can be acquired when the number of apertures is larger than 20. In our experiment, the number of apertures was set to 33; other numbers are also feasible. With the number of apertures set at 33, an experiment was performed to test the calibration accuracy of our method in a laboratory held at constant temperature. In addition, the turntable used in the experiments was placed on a gas-floating vibration isolation platform to avoid vibrational disturbances. We tested the calibration accuracy of the principal distance and the principal point while making multiple measurements. We processed tens of thousands of images to determine the calibration accuracy. The variation of the principal distance using images collected within a time interval of 2000 s was determined. Using the data, the mean square error was computed and the variation of the principal distance was determined to be as high as 0.017 mm. The variation in the coordinates of the principal point reached 0.005 mm and 0.0049 mm in the *x* and *y* directions, respectively. The camera has 5 m image positioning accuracy. It requires less than 50 μm calibration accuracy of the principal distance and the principal point. The variability was at the micron level and met the camera mapping requirements.

We also compared our method with other calibration methods, such as self-calibration^25^, orientation model^26^, arbitrary coplanar circles^27^ and angle measurement, for calibration accuracy, processing speed and maneuverability. We also tested the calibration precision of conventional measurement angle-based methods using a single point emitted by a collimator. The calibration precision of principal calibration methods for remote sensing cameras has been reported in Refs. 25, 26. Table 2 summarizes the comparisons among the proposed method and other methods. The calibration precision of this method is seen to be better than that of other methods.

Our method uses 12 images to perform each calibration. The bundle adjustment method uses 200 images to perform each calibration^28^. For a complete calibration, the test field method and the goniometric method need ~20 images^29^. The angle measurement method requires more than 100 images. Our method requires the smallest number of images, which means that the method consumes the smallest amount of time for each calibration. In addition, our method can perform a calibration without the use of a reference distance. We basically utilized the same calibration set-up as the angle measurement method. As mentioned earlier, self-calibration methods usually require several reference test fields to be viewed from different angles. Other methods, such as the 180° maneuvering method^30^ and ground control point (GCP)-based method^31^, usually require many external reference targets. Therefore, our proposed method simplifies the calibration process, is easy to operate and requires less auxiliary equipment and computation time for calibrating remote sensing cameras.

## Conclusions

In this paper, we propose a MEMS-LLAD for use in the collimator used for calibrating optical cameras to improve calibration accuracy. The traditional optical focal plane of the collimator typically uses a single light lead-in array. As a result, calibration accuracy is generally unreliable. We prepared an MEMS-LLAD for use at the optical focal plane of the collimator. Without additional volume or power consumption, the random error of this calibration system was decreased using a multi-image matrix.

## Figures and Tables

**Table 1:** Calculated results for the principal distance and principal point using the MEMS-LLAD and the SHLL methods.

Number	Elements	Calibrated value	Reference value
1	f (mm)	2032.0882	2032.2812
2	U0*x* (mm)	–0.5660	–0.8223
3	U0*y* (mm)	–0.9528	–0.9828

Abbreviations: LLAD, light lead-in array device; MEMS, micro-electro-mechanical-system; SHLL, single-hole light lead-in.

**Table 2:** Comparisons of calibration precision of different methods.

Methods	Principal distance	Principal point
Self-calibration^25^	8.164 mm	0.0039 mm
Orientation model ^26^	18.9 pixels	1.3 pixels
Arbitrary coplanar circles^27^	9.21 pixels	—
Angle measurement	0.0189 mm (3.47 pixels)	0.0067 mm (0.32 pixels)
Our method	0.017 mm (3.11 pixels)	0.005 mm (0.28 pixels)
